# Diagnostic Yield of a Direct Quantitative Smear of Lower Respiratory Tract Secretions in Patients with Suspected Pneumonia Compared to a Semi-quantitative Culture

**Published:** 2017

**Authors:** Shervin Shokouhi, Ilad Alavi Darazam, Maryam Sadeghi, Latif Gachkar, Samaneh Dolatshahi

**Affiliations:** Infectious Diseases and Tropical Medicine Research Center, Shahid Beheshti University of Medical Sciences, Tehran, Iran

**Keywords:** Semi-quantitative culture, Bacteria, Pneumonia, Bronchoalveolar lavage, Intensive care units

## Abstract

**Background:**

Microorganism isolation from respiratory tract specimens is the standard of care in patients with suspected nosocomial and ventilator associated pneumonia. However, these methods are time-consuming and are influenced by several factors. A direct quantitative smear (DQS) with proper staining may be an easy, cost-effective, rapid method. We evaluated the diagnostic yield of direct smears compared to semi-quantitative culture methods.

**Materials and Methods:**

Hospitalized, intubated patients with clinically suspected pneumonia and patients who underwent diagnostic bronchoscopic alveolar lavage (BAL) and trans-endotracheal aspiration (TEA) were enrolled in a prospective study. The obtained specimens were Gram stained and microorganisms were computed per 10 high-power fields (HPFs) of light microscopy. All samples were cultured by a standard semi-quantitative method. Colony-forming units (CFU) >10^4^/mL and >10^5^ CFU/mL were reported as culture-positive for BAL and TEA, respectively.

**Results:**

A total of 331 respiratory specimens were analyzed. Based on culture results, the best cut-off point was 35 microorganisms in 10 HPFs of microscopy and provided 90.4% sensitivity and 90.8% specificity. The best cut-off point for 25 microorganisms in 10 fields of light microscopy provided 95.2% sensitivity and 85.7% specificity.

**Conclusion:**

A DQS obtained by BAL and TEA may be a reliable and rapid method to diagnose pneumonia and anticipate semi-quantitative culture results. The sensitivity and specificity of a direct smear have adequate diagnostic yield to recommend it as an adjunct to microorganism-isolation methods.

## INTRODUCTION

Pneumonia is among the 10 common causes of mortality among all age groups, and is the most common cause of infectious mortality. Appropriate and early treatment according to reliable guidelines can decrease mortality due to infection; antibiotic therapy should be started immediately after diagnosis (1).

The diagnosis with the highest specificity and sensitivity can be made through observation of clinical signs and symptoms and evaluation of sputum smear and culture together. To differentiate bacterial colonization from a respiratory tract infection, the cut-off points for the numbers of bacteria in respiratory secretion cultures were determined based on the methods used to obtain the secretion. For respiratory trachea cultures, a cut-off point of 10^5^ colony/mL was considered significant to indicate an infection; 10^4^ colony/mL was considered significant for bronchoalveolar lavage (BAL) fluid cultures (1,2).

In a study by Miyashita et al. of methods to determine the reliability of a sputum smear and culture to diagnose community-acquired pneumonia, respiratory secretion samples were evaluated based on smears and cultures (2). However, smear evaluations only reported the morphological properties of the microorganism (cocci, bacilli, Gram-positive or -negative) and its dominance (without providing a definition from dominance) and compared those with the culture results (1). In a review by Reed et al. to determine the importance of Gram staining to diagnose pneumococcal pneumonia, no unique estimation was made to evaluate the accuracy and specificity of this method (3). It was also recommended to consider purulent sputum samples with 10 microorganisms in each microscopic field as positive (1).

Considering the progressive increase in antimicrobial resistance, the proper treatment based on the type of microorganism should be initiated to prevent the indiscriminate use of antibiotics. However, microbial culture testing is a time-consuming procedure while smear evaluations are provided within hours. Therefore, the current study evaluated respiratory secretions in patients suspected of pneumonia to determine the cut-off point for the number of bacteria in the microscopic field of a smear compared with the results of culture testing as a possible guide for antibiotic administration.

## MATERIALS AND METHODS

In the current study, samples from the lower respiratory tract were obtained from patients in the intensive care unit (ICU) or other wards who were intubated due to different causes (such as trauma, low consciousness, post-operative care, respiratory distress, sepsis, or severe pneumonia); and had increasing respiratory secretions (clear, white, or purulent) or fever (>38.5°C) or new changes in radiography images (scattered or localized infiltration) or all of them. Respiratory secretion specimens were collected through tracheal aspiration after adding 5 mL of normal saline into the tracheal tube of the patient using a sterile catheter, and were immediately transferred to the laboratory in sterile tubes. BAL specimens collected from patients who underwent bronchoscopy due to different reasons (respiratory infectious/non-infectious diseases) were also transferred to the laboratory in sterile tubes.

Samples were evaluated under optical microscopy (×100) after Gram staining and those with >25 neutrophils and <10 epithelial cells (1) were re-evaluated for bacterial counts in 10 microscopic fields. The quantitative smear analysis results were reported as microorganisms/10 high-power fields (HPFs). Specimens were incubated 48–72 h and the results were assessed and reported based on colony count per milliliter. Contaminated smears and cultures with Candida spp. were excluded. Two experts analyzed all experiments. A semi-quantitative culture of >10^5^ colony/mL for tracheal aspiration specimens and >10^4^ colony/mL for BAL samples was considered significant (3).

Specimens were then divided into two groups (tracheal aspiration and BAL); results of the quantitative smear analysis of each group were compared with corresponding semi-quantitative cultures (gold standard), and the receiver operating characteristic (ROC) curve was used to determine the sensitivity and specificity at different cut-off points.

## RESULTS

In the current study, 244 patients suspected of pneumonia (171 men and 73 women) with a mean age of 64.3 ± 18.7 years (range, 12–96 years) were evaluated. The cultures of lower respiratory tract specimens were positive in 125 samples. The mean number of microorganisms reported in 10 fields of optical microscopy was 47 ± 38 (range, 0–100); there were 5 samples with 0 microorganisms in smear analysis.

Using an ROC curve, the best cut-off point was 35 microorganisms in 10 fields of optical microscopy and provided 90.4% sensitivity and 90.8% specificity. The best cut-off point for 25 microorganisms in 10 fields of optical microscopy provided 95.2% sensitivity and 85.7% specificity. The accuracy of the model for both cut-off points (area under the curve, AUC) was estimated to be 95.7% (P < 0.001).

For BAL samples, 33 samples obtained from patients suspected of pneumonia (15 men and 18 women) with a mean age of 59.5 ± 20.2 years (range 13–96 years) were evaluated. The culture test result was positive for 22 samples. The mean number of reported microorganisms for 10 fields of optical microscopy was 28.7 ± 34.4 (range 0–100); there were 2 samples with 0 microorganisms in the smear test. Using an ROC curve, the best cut-off points for 9 microorganisms in 10 fields of optical microscopy provided 86.4% sensitivity and 81.2% specificity; the best cut-off points for 6.5 microorganisms in 10 fields of optical microscopy provided 86.4% sensitivity and 72.7% specificity. The accuracy of the model for cut-off points (AUC) was estimated to be 90.5% (P < 0.001).

Most of the microorganisms isolated were *Acinetobacter* spp. (34.3%), *Pseudomonas* spp. (15.3%), *Staphylococcus aureus* (13.4%), and miscellaneous (36.1%), respectively.

## DISCUSSION

This study aimed to indicate the use of smear evaluation to identify pneumonia; accordingly, the cut-off point for the number of microorganisms in the smear was provided in proportion and appropriate to the results of culture testing. To increase the accuracy of the study, the number of microorganisms in 10 fields of optical microscopy was counted. Results of the current study provided closer and more specific cut-off points compared with those of previous studies (25–35 microorganisms in samples obtained from tracheal suction and 6.5–9 microorganisms in samples obtained from BAL fluids with acceptable specificity and sensitivity). The study results also confirmed the diagnostic value of the smear provided in some previous studies.

In a study conducted by Mimoz et al. on the diagnostic value of smears to diagnose ventilator-associated pneumonia (VAP) through invasive methods such as protected specimen brushes and plugged telescopic catheters, respiratory secretions were collected and evaluated (4). Similar to the current study, they also counted the microorganisms in several fields of optical microscopy to increase the accuracy of the study. The results were then compared to those of culture testing. There was a significant relationship between the presence of bacteria in the smear and the final result of culture testing (74%–81% sensitivity and 94%–100% specificity), but no cut-off value was provided for the smear test results and only the specificity and sensitivity of the smear test, compared with those of the culture test, were emphasized.

According to the culture results of tracheal fluids collected through suctioning of intubated ICU patients (with/without susceptibility to pneumonia), the cut-off point was provided for the number of intracellular microorganisms in the smear test. Approximately 300 neutrophils were evaluated in each sample. In the cut-off points of 5% and 7%, the sensitivity values of 85% and 61% and specificity values of 82% and 91% were provided (5), respectively; their results had lower sensitivity and similar specificity values compared with those of the current study. The difference between cut-off values in the two studies may result from different methods employed to count microorganisms in the smears. In the study by Brasel et al., the number of intracellular microorganisms was evaluated in 300 neutrophils, but the number of microscopic fields these neutrophils were counted in was not clear (5).

In the study by Solé-Violán et al., samples were collected from 33 patients who were hospitalized in the ICU, intubated for more than three days, and susceptible to pneumonia based on the clinical and radiological signs and symptoms; samples were collected through BAL and PBS methods. Sensitivity and specificity for BAL fluid and PBS culturing, and the number of intracellular microorganisms in the smear, were used to diagnose pneumonia; the sensitivity and specificity values of 62% and 100%, respectively, were provided at the aforementioned cut-off points (6).

The sensitivity and specificity values of 86.4% and 72.7%–81.2%, respectively, were provided for the cut-off points of 6.5–9 microorganisms in BAL fluid samples.

The primary reason for the difference in sensitivity and specificity of the current study may result from different standards and scales to compare smear results. A diagnosis of pneumonia can be achieved through autopsy, blood or effusion pleural fluid culturing, cavity formation in radiographic images, or clinical response to proper antibiotic therapy. In the current study, the smear evaluations were compared with the results of positive or negative cultures. In addition, 4% of the positive smear was approximately set based on the variables assessed in patients with/without infection. It is noteworthy that in the current study, the number of microorganisms in 300 neutrophils was counted for each sample.

Another study by Allaouchiche et al. included patients admitted to the ICU, intubated, and suspected of pneumonia based on clinical and radiographic findings. The patients underwent BAL and PBS; the collected samples were primarily evaluated through smear testing and then culture testing, and a diagnosis of pneumonia was determined using PBS culture results and clinical improvement after administration of proper antibiotics. The sensitivity and specificity of the number of intracellular microorganisms in 100 cells (macrophages and neutrophils) with a cut-off point of 2% for early diagnosis of VAP were evaluated, and the results showed 86.3% sensitivity and 78.9% specificity (7).

## CONCLUSION

Use of smears with a significant number of bacteria that is sufficient to diagnose pneumonia is a suitable method with reliable sensitivity and specificity, which can be used to make a prompt diagnosis of pneumonia before the before culture results are available.

Further studies are necessary to provide different cut-off points based on the type of microorganism in the smear and to determine their sensitivity and specificity.

## References

[1] Bennett JE Dolin R Blaser MJ. Mandell, Douglas, and Bennett’s principles and practice of infectious diseases. Elsevier Health Sciences; 2014 9 2.

[2] Miyashita N Shimizu H Ouchi K Kawasaki K Kawai Y Obase Y Assessment of the usefulness of sputum Gram stain and culture for diagnosis of community-acquired pneumonia requiring hospitalization. Med Sci Monit 2008;14(4):CR171–6. 18376343

[3] Reed WW Byrd GS Gates RH Jr Howard RS Weaver MJ. Sputum gram’s stain in community-acquired pneumococcal pneumonia. A meta-analysis. West J Med 1996;165(4):197–204. 8987424 PMC1303744

[4] Mimoz O Karim A Mazoit JX Edouard A Leprince S Nordmann P. Gram staining of protected pulmonary specimens in the early diagnosis of ventilator-associated pneumonia. Br J Anaesth 2000;85(5):735–9. 11094590 10.1093/bja/85.5.735

[5] Brasel KJ Allen B Edmiston C Weigelt JA. Correlation of intracellular organisms with quantitative endotracheal aspirate. J Trauma 2003;54(1):141–4; discussion 144–6. 12544909 10.1097/00005373-200301000-00017

[6] Solé-Violán J Rodríguez de Castro F Rey A Martín-González JC Cabrera-Navarro P. Usefulness of microscopic examination of intracellular organisms in lavage fluid in ventilator-associated pneumonia. Chest 1994;106(3):889–94. 8082373 10.1378/chest.106.3.889

[7] Allaouchiche B Jaumain H Chassard D Boulétreau P. Gram stain of bronchoalveolar lavage fluid in the early diagnosis of ventilator-associated pneumonia. Br J Anaesth 1999; 83(6):845–9. 10700780 10.1093/bja/83.6.845

